# Supplementary material for: CD200–CD200R immune checkpoint engagement regulates ILC2 effector function and ameliorates lung inflammation in asthma
Source: Nat Commun. 2021 May 5;12:2526. doi: 10.1038/s41467-021-22832-7 (PMC8100131; doi:10.1038/s41467-021-22832-7)
Supplement: Supplementary file 1 — Supplementary Information [file 41467_2021_22832_MOESM1_ESM.pdf]

CD200–CD200R immune checkpoint engagement regulates ILC2 effector function and ameliorates lung inflammation in asthma

Shafiei-Jahani et. al, Supplementary information.

Supplementary Figure 1

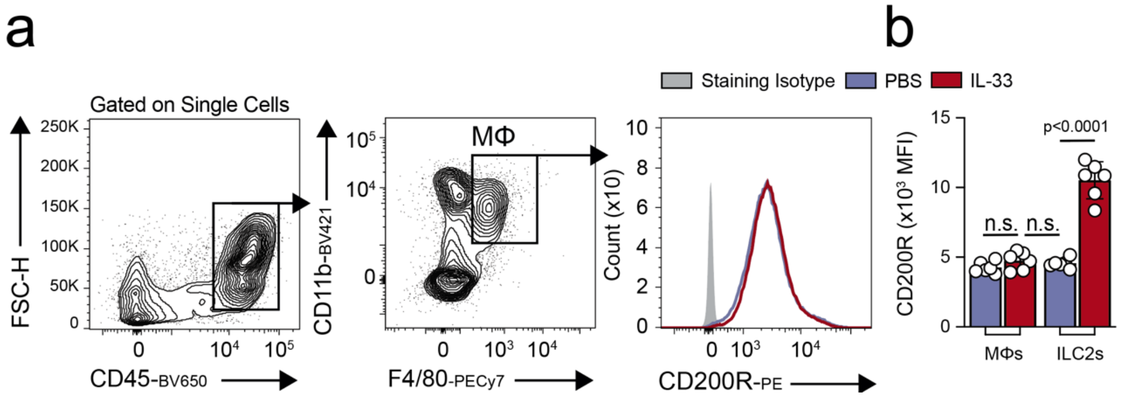

CD200R expression on macrophages and ILC2s in the lungs. A cohort of WT mice were challenged with recombinant mouse IL-33 or PBS intranasally (i.n.) on days 1, 2 and 3. The mice were euthanized on day 4 and the lung was analyzed by flow cytometry. **(a)** macrophages gated as $CD45^{+}CD11b^{+}F4/80^{+}$ cells **(b)** CD200R expression on macrophages and ILC2s (Lin^{-} $CD45^{+}CD127^{+}ST2^{+}$) in the lungs. Data are shown as means ± SEMs and are representative of 3 individual experiments. Statistical analysis, one-way ANOVA.

Supplementary Figure 2

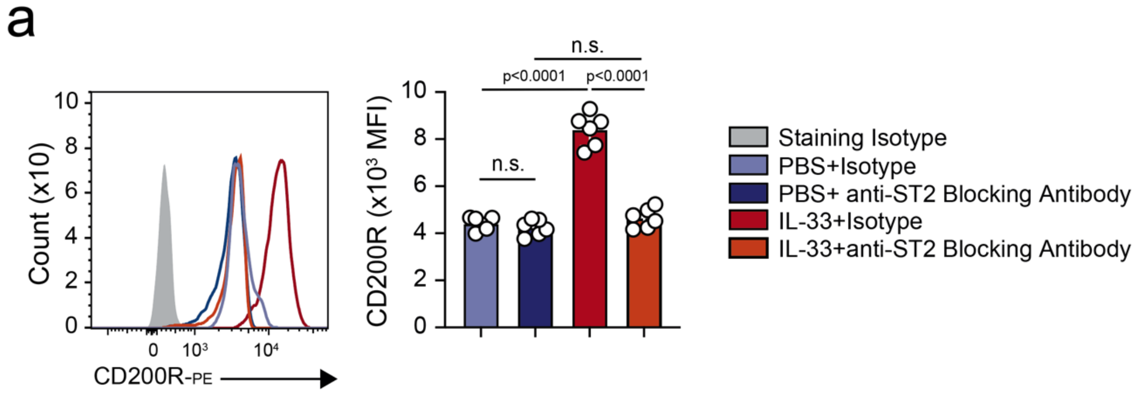

CD200R induction is dependent on ST2 engagement by IL-33. Freshly sorted naïve pulmonary ILC2s were cultured them in presence of IL-2, IL-7, and *ex vivo* stimulated with IL-33 or PBS, n=6 mice. Additionally, the cells were treated with either anti-ST2 blocking antibody or isotype control. **(a)** CD200R expression after 48 hours. Data are shown as means ± SEMs and are representative of 3 individual experiments. Statistical analysis, one-way ANOVA.

Supplementary Figure 3

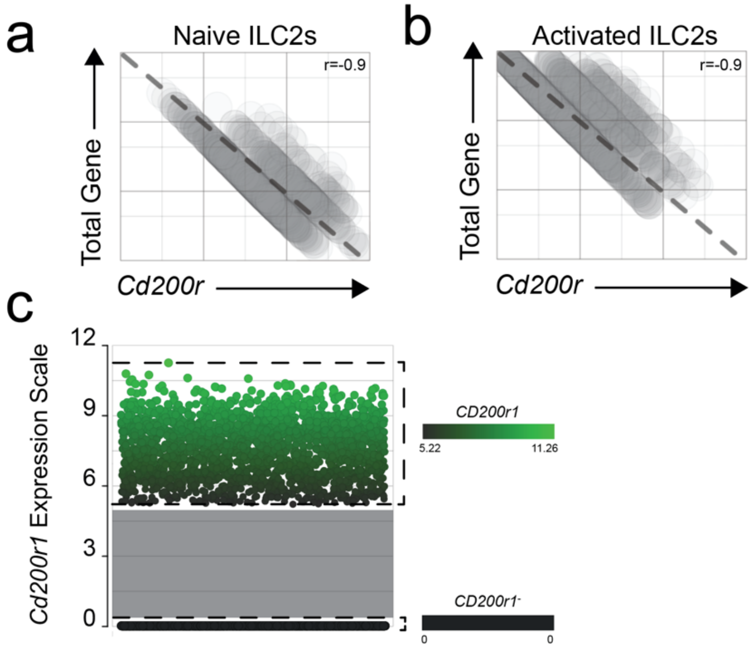

CD200R expression is negatively correlated with total gene expression. Correlation plots depicting CD200R mRNA expression vs. total gene expression in **(a)** naïve and **(b)** IL-33 activated ILC2s. **(c)** CD200R expression landscape. Dots represent individual cells.

Supplementary Figure 4

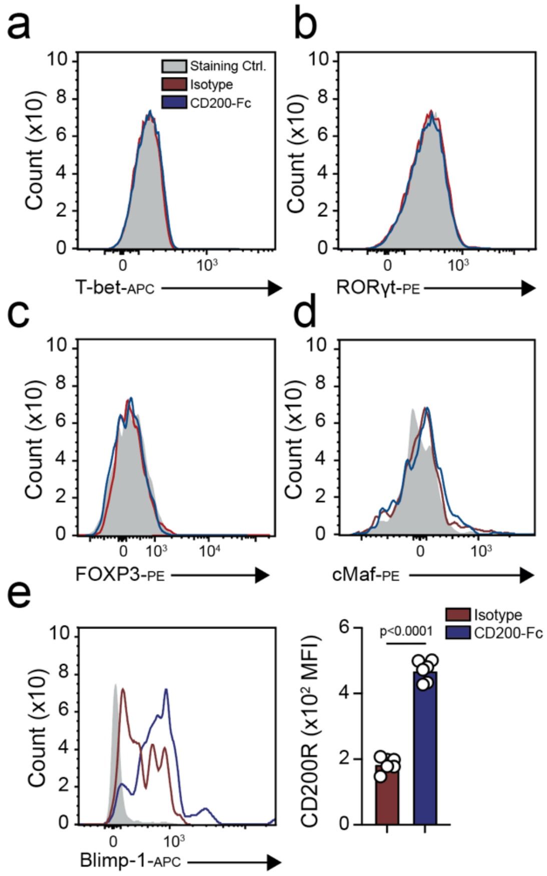

CD200R engagement on ILC2s induces Blimp-1. IL-33-activated pulmonary ILC2s were freshly sorted and cultured *ex vivo* with either CD200-Fc or isotype control for 24 hours, n=6 mice. The cells were subsequently analyzed by flow cytometry. Expression levels of **(a)** T-bet, **(b)** RORγt, **(c)** FOXP3, **(d)** cMaf, and **(e)** Blimp-1. Data are shown as means ± SEMs and are representative of 3 individual experiments. Statistical analysis, two-tailed student's t-test.

Supplementary Figure 5

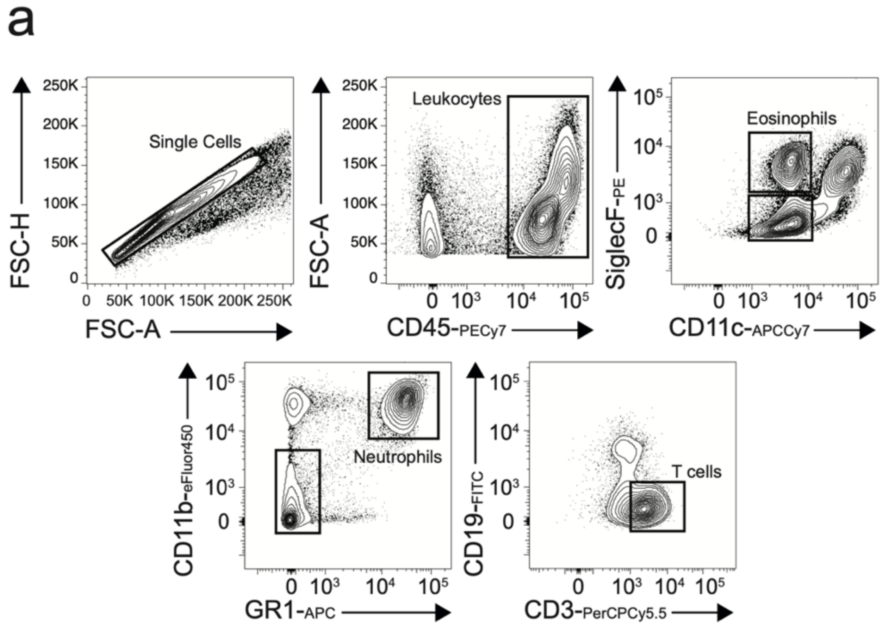

Gating strategy of BAL fluid. (a) leukocytes are defined as all $CD45^+$ cells. Eosinophils are gated as $CD45^+CD11c^-SiglecF^+$ cells. Neutrophils are gated $CD45^+CD11c^-SiglecF^-Gr\text{-}1^-CD11b^+$ cells. T cells are defined as $CD45^+CD11c^-SiglecF^-Gr\text{-}1^-CD11b^-CD19^-CD3^+$ cells.

Supplementary Figure 6

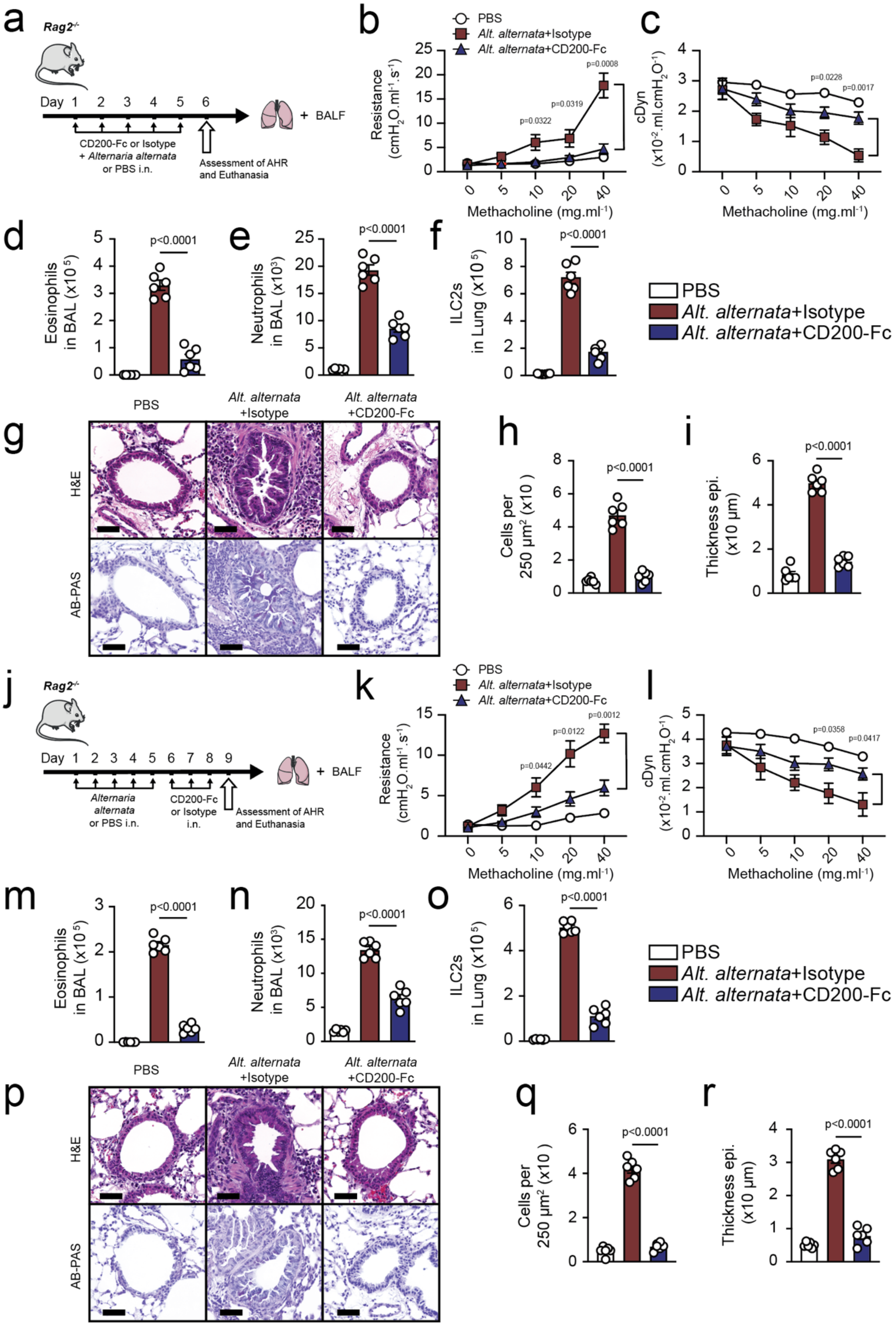

CD200R engagement ameliorates *Alternaria alternata*-induced AHR. (a) A cohort of *Rag2* $^{-/-}$ mice were treated with CD200-Fc *i.n.* or isotype control and were challenged with *Alternaria alternata* (100 µg in 50 µL) or PBS intranasally (*i.n.)* on days 1 to 5. On day 6, we assessed the lung function as shown in the timeline, n=6 mice **(b** and **c)** Line graph show lung resistance and dynamic compliance (cDyn) in response to increasing doses of methacholine. **(d** and **e)** the numbers of eosinophils and neutrophils in the BAL. **(f)** the numbers of ILC2s in the lungs. (**g–i)** Representative images and quantification of H&E and AB-PAS stained histologic sections of the lungs. Scale bars=50 µm. **(j)** A cohort of *Rag2* $^{-/-}$ mice were challenged with *Alternaria alternata* (100 µg in 50 µL) or PBS intranasally (*i.n.)* on days 1 to 5, n=6 mice. Subsequently, the mice were treated with CD200-Fc *i.n.* or isotype control on days 6, 7, and 8. The lung function and samples were measured on day 9. **(k)** lung resistance. **(l)** dynamic compliance. **(m** and **n)** number of eosinophils and neutrophils in BAL fluid. **(o)** number of ILC2s in lungs. Histological images of the lungs **(p)** and corresponding quantifications of cell numbers **(q)** and thickness of epithelium **(r).** Scale bars=50 µm. Data are shown as means ± SEMs and are representative of 3 individual experiments. Statistical analysis, two-tailed student's t-test. Mouse and lung images are provided with permission from Servier Medical Art.
