# Supplementary material for: CD200–CD200R immune checkpoint engagement regulates ILC2 effector function and ameliorates lung inflammation in asthma
Source: Nat Commun. 2021 May 5;12:2526. doi: 10.1038/s41467-021-22832-7 (PMC8100131; doi:10.1038/s41467-021-22832-7)
Supplement: Supplementary file 3 — Reporting Summary [file 41467_2021_22832_MOESM3_ESM.pdf]

nature research

Corresponding author(s): Omid Akbari
Last updated by author(s): 2/18/2021

Reporting Summary

Nature Research wishes to improve the reproducibility of the work that we publish. This form provides structure for consistency and transparency in reporting. For further information on Nature Research policies, see Authors & Referees and the Editorial Policy Checklist .

Statistics

For all statistical analyses, confirm that the following items are present in the figure legend, table legend, main text, or Methods section.

n/a	Confirmed	
☐	☒	The exact sample size (*n*) for each experimental group/condition, given as a discrete number and unit of measurement
☐	☒	A statement on whether measurements were taken from distinct samples or whether the same sample was measured repeatedly
☐	☒	The statistical test(s) used AND whether they are one- or two-sided<br>*Only common tests should be described solely by name; describe more complex techniques in the Methods section.*
☒	☐	A description of all covariates tested
☒	☐	A description of any assumptions or corrections, such as tests of normality and adjustment for multiple comparisons
☐	☒	A full description of the statistical parameters including central tendency (e.g. means) or other basic estimates (e.g. regression coefficient) AND variation (e.g. standard deviation) or associated estimates of uncertainty (e.g. confidence intervals)
☐	☒	For null hypothesis testing, the test statistic (e.g. *F*, *t*, *r*) with confidence intervals, effect sizes, degrees of freedom and *P* value noted<br>*Give P values as exact values whenever suitable.*
☒	☐	For Bayesian analysis, information on the choice of priors and Markov chain Monte Carlo settings
☒	☐	For hierarchical and complex designs, identification of the appropriate level for tests and full reporting of outcomes
☒	☐	Estimates of effect sizes (e.g. Cohen's *d*, Pearson's *r*), indicating how they were calculated

Our web collection on statistics for biologists contains articles on many of the points above.

Software and code

Policy information about availability of computer code

Data collection	BD FACSDiva software v8.0.1 was used for flow cytometry data aquisition
Data analysis	Partek Genomics Suite software, version 7.0 Copyright, FlowJo software (TreeStar) version 10, ImageJ 1.4 , Prism analysis software version 5-8, MasterPlex2012 software, Adobe Illustrator software 2018-2021.

For manuscripts utilizing custom algorithms or software that are central to the research but not yet described in published literature, software must be made available to editors/reviewers. We strongly encourage code deposition in a community repository (e.g. GitHub). See the Nature Research guidelines for submitting code & software for further information.

Data

Policy information about availability of data

All manuscripts must include a data availability statement. This statement should provide the following information, where applicable:
- Accession codes, unique identifiers, or web links for publicly available datasets
- A list of figures that have associated raw data
- A description of any restrictions on data availability

Sequence data that support the findings of this study have been deposited in Genbank with the primary accession code GSE166889. All remaining data will be made available by the corresponding author upon reasonable request.

Field-specific reporting

Please select the one below that is the best fit for your research. If you are not sure, read the appropriate sections before making your selection.

☒

Life sciences study design

All studies must disclose on these points even when the disclosure is negative.

Sample size	We used ANOVA to calculate the sample size with the assumption of power of 80%, effect size of ~~50, and alpha less than 0.05.
Data exclusions	No animals and other data were excluded. There was no anticipation of excluding animals once they underwent the experiments.
Replication	All attempts at replication were successful. All data are representative of at least three independent experiments.
Randomization	Age-matched male mice were randomly allocated to experimental groups. Randomization process consisted of pooling all the mice in a large cage and random picking out and allocating to experimental groups. All healthy human subjects were blindly and randomly allocated to experimental groups.
Blinding	No blinding was done for experiments as the design and conditions prevent possible sources of bias.

Reporting for specific materials, systems and methods

We require information from authors about some types of materials, experimental systems and methods used in many studies. Here, indicate whether each material, system or method listed is relevant to your study. If you are not sure if a list item applies to your research, read the appropriate section before selecting a response.

Materials & experimental systems

n/a	Involved in the study	
☐	☒	Antibodies
☒	☐	Eukaryotic cell lines
☒	☐	Palaeontology
☐	☒	Animals and other organisms
☐	☒	Human research participants
☒	☐	Clinical data

Methods

n/a	Involved in the study	
☒	☐	ChIP-seq
☐	☒	Flow cytometry
☒	☐	MRI-based neuroimaging

Antibodies

Antibodies used

Anti-mouse antibodies
Biotin (supplier: BioLegend; cat. #: 100304; clone: 145-2C11; lot #: B216147)
Biotin - CD45R/B220 (supplier: BioLegend; cat. #: 103204; clone: RA3-6B2; lot #: B226660)
Biotin - Gr-1 (supplier: BioLegend; cat. #: 108404; clone: RB6-8C5; lot #: B200655)
Biotin - CD11c (supplier: BioLegend; cat. #: 117304; clone: N418; lot #: B217819)
Biotin - CD11b (supplier: BioLegend; cat. #: 101204; clone: M1/70; lot #: B221905)
Biotin - Ter119 (supplier: BioLegend; cat. #: 116204; clone: TER-119; lot #: B218645)
Biotin - FceRIa (supplier: BioLegend; cat. #: 134304; clone: MAR-1; lot #: B218942)
Biotin - TCR-gd (supplier: eBioscience; cat. #: 13-5711-85; clone: eBioGL3; lot #: 4274982)
Biotin - CD5 (supplier: BioLegend; cat. #: 100604; clone: 53-7.3, lot #: B202966)
Biotin-NK1.1 (supplier: BioLegend; cat. #: 108704; clone: PK136)
FC-block (supplier: BioXcell, cat. #: BE0307 clone: 2.4G2)
FITC - Streptavidin (supplier: BioLegend; cat. #: 405202; lot #: B190908)
PE-Cy7 - CD127 (supplier: BioLegend; cat. #: 135014; clone: A7R34; lot #: B222850)
APCCy7 - CD45 (supplier: BioLegend; cat. #: 103116; clone: 30-F11; lot #: B237400)
PerCP - eFluor710-ST2 (supplier: eBioscience; cat. #: 46-9335-82; clone: RMST2-2; lot #: 4300549)
PE - IgG2B (supplier: R&D Systems; clone: 133303, cat. #: IC0041P; lot #: LHG1917021)
PE – Rabbit Hamster IgG (supplier: R&D Systems; clone: 60024B, cat. #: IC1051P)
APC-IgG2a kappa (supplier: Thermofisher; cat. #: 17-4321-81, clone: eBR2a)
PE- IgG2a kappa (supplier: Thermofisher; cat. #: 12-4031-82, clone: eB149/10H5)
eFluor 450- IgG1 kappa (supplier: Thermofisher; cat. #: 48-4301-82, clone: eBRG1)
PE- IgG1, κ (supplier: BioLegend; cat. #: 400408, clone: RTK2071)
PE-IgG XP (supplier: Cell Signaling Technology; cat. #: 5742S, clone: DA1E)
PE/Cy7 - CD45 (supplier: BioLegend; cat. #: 103114; clone: 30-F11; lot #: B219150)
APC/Cy7 - CD11c (supplier: BioLegend; cat. #: 117324; clone: N418; lot #: B237079)
PE-Siglec-F (supplier BD Bioseinces; cat.#: 552126; clone: E50-2440; lot #:5343971)
APC-Gr1 (supplier: BioLegend; cat.#: 108412, clone: RB6-8C5, lot #: B236640)
eFluor450 - CD11b (supplier: eBioscience; cat. #: 48-0112-82; clone: M1/70; lot #: 4289827)
PerCP Cy5.5-CD3e (supplier: BioLegend; cat. #:100218, clone: 17A2)

FITC-CD19 (supplier: BioLegend; cat. #: 115506, clone: 6D5)
PE- CD200r (supplier: Sino Biological; cat. #: 50209-R181-P; clone: 181)
APC-Ki-67 (supplier: Thermofisher; cat. #: 17-5698-82, clone: SolA15)
PE-GATA3 (supplier: Thermofisher; cat. #: 12-9966-42, clone: TWAJ)
eFluor 450-anti-mouse IL-13 (supplier: Thermofisher; cat. #: 48-7133-82, clone: eBio13)
PE-anti-mouse/human IL-5 (supplier: BioLegend; cat. #: 504304, clone: TRFK5)
PE-Phospho-IKKα/β (supplier: Cell Signaling Technology; cat. #: 14938, clone: 16A6)
Alexa Fluor 647 - NFκB p52 (supplier: Santa Cruz Biotechnology; cat. #: sc-7386; lot #: J1311)
PE - RelA NFκB p65 (supplier: R&D Systems; cat. #: IC5078P; lot #: ADVN0113101)
BV650-CD45 (supplier: BioLegend; cat. #: 103151, clone: 30-F11)
BV421-CD11b (supplier: BioLegend; cat. #: 101236, clone: M1/70)
PECy7-F4/80 (supplier: BioLegend; cat. #: 123114, clone: BM8)
ST2 blocking antibody (supplier: BioLegend; cat. #: 146604, clone: DIH4)
Rat IgG1, κ Isotype Ctrl Antibody (supplier: BioLegend; cat. #: 400402, clone: RTK2071)
APC-T-bet (supplier: Thermofisher; cat. #: 17-5825-82, clone: eBio4B10 (4B10))
PE-RORγt (supplier: Thermofisher; cat. #: 12-6988-82, clone: AFKJS-9)
PE-FOXP3 (supplier: BioLegend; cat. #: 126404, clone: MF-14)
PE-cMaf (supplier: Thermofisher; cat. #: 12-9855-42, clone: sym0F1)
APC-Blimp-1 (supplier: BioLegend; cat. #: 150008, clone: 5E7)

Anti-human antibodies
FITC - Lineage, includes mixture of CD3, CD14, CD16, CD19, CD20, CD56 (supplier: BioLegend; cat. #: 348801; lot #: B215228)
FITC - CD235a (supplier: BioLegend; cat. #: 349104; clone: HI264; lot #: B251984)
FITC - FCeRIa (supplier: BioLegend; cat. #: 334608; clone: AER-37; lot #: B226717)
FITC - CD1a (supplier: BioLegend; cat. #: 300104; clone: HI149; lot #: B155264)
FITC - CD123 (supplier: BioLegend; cat. #: 306014; clone: 6H6; lot #: B167088)
FITC - CD5 (supplier: BioLegend; cat. #: 364022; clone: L17F12; lot #: B256486)
APCCy7 - CD45 (supplier: BioLegend; cat. #: 304014; clone: HI30; lot #: B214034)
BV421 – Cd200r (supplier: BioLegend; cat. #: 329314, clone: OX-108)
BV421 – IgG1,k (supplier: BioLegend; cat. #: 400158; clone: MOPC-21)
PE - CD294 (CRTH2) (supplier: BioLegend; cat. #: 350106; clone: BM16; lot #: B179655)
PE/Cy7 - CD127 (IL-7Ra) (supplier: BioLegend; cat. #: 351320; clone: A019D5; lot #: B191896)

Validation

All the antibodies are from commercial source and have been validated by the vendors and their validation data are available on the manufacturers' website (BioLegend, Thermofisher, R&D Systems, Santa Cruz Biotechnology, Cell Signaling Technology, and eBioscience). Manufacturers' datasheets that provide quality testing methods, validated applications, reported applications, usage instructions, application notes, publications using the reagent, and other relevant data.

Animals and other organisms

Policy information about studies involving animals; ARRIVE guidelines recommended for reporting animal research

Laboratory animals

We used 5-8 weeks old female mice. Strains include: BALB/cByJ (JAX stock number: 000651), RAG2 deficient (C.B6(Cg)-Rag2tm1.1Cgn/J) (JAX stock number: 008448), RAG2-, IL-2Rg-deficient (C;129S4-Rag2tm1.1Flv Il2rgtm1.1Flv/J) (JAX stock number: 014593) were purchased from the Jackson Laboratory (Bar Harbor, Maine). Mice were maintained at macroenvironmental temperature of 21-22°C, humidity (48-52%), in a conventional 12:12 light/dark cycle with lights on at 6:00 a.m. and off at 6:00 p.m.

Wild animals

No wild animals were used in this study.

Field-collected samples

No field-collected samples were used in this study.

Ethics oversight

All the experiments described in this manuscript were approved IACUC of USC and in complete compliance with the guidelines of IACUC of USC. Animal facilities at the USC are AAALAC accredited.

Note that full information on the approval of the study protocol must also be provided in the manuscript.

Human research participants

Policy information about studies involving human research participants

Population characteristics

Human blood samples were obtained from male and female healthy donors (Age 18 to 65). Blood obtained from subjects were randomly assigned to different experimental groups.

Recruitment

No specific selection criteria was applied for healthy donors. Healthy participants were randomly recruited and randomly allocated to different experimental groups to minimize any biases.

Ethics oversight	All human studies were approved by USC Institutional review board and conducted in accordance to the principles of the Declaration of Helsinki. Informed consent was obtained from all subjects according to our approved IRB protocols .

Note that full information on the approval of the study protocol must also be provided in the manuscript.

Flow Cytometry

Plots

Confirm that:

- [x] The axis labels state the marker and fluorochrome used (e.g. CD4-FITC).
- [x] The axis scales are clearly visible. Include numbers along axes only for bottom left plot of group (a 'group' is an analysis of identical markers).
- [x] All plots are contour plots with outliers or pseudocolor plots.
- [x] A numerical value for number of cells or percentage (with statistics) is provided.

Methodology

Sample preparation	This information is included in the Methods section. Briefly, utilizing fine surgical scissors, murine lungs were surgically removed and minced in a sterile environment subsequently incubated in type IV collagenase (1.6 mg/mL; Worthington Biochemicals, Lakewood, NJ) at 37°C for 60 minutes. After digestion, murine lung fragments were then pressed through a 70 µm nylon cell strainer, using the rubber end of a sterile 10 mL syringe plunger, in order to create a single cell suspension. In order to terminate the enzymatic reaction of collagenase, the cells were washed with 1x phosphate buffered saline (PBS) by centrifugation at 400x g for 7 minutes at 4°C. In order to exclude and lyse the red blood cells (RBCs), the cell pellet was subsequently resuspended in 1x RBC lysis buffer (Biolegend®, San Diego, CA) and incubated at room temperature (RT) for 5 minutes. In order to terminate the chemical reaction, the cells were subsequently washed and centrifuged—at 400x g for 7 minutes at 4°C—with 1x PBS.
Instrument	Stained cells were analyzed on FACSCanto II and/or FACSARIA III systems (Becton Dickinson)
Software	The software used to collect samples is BD Facsdiva and the data were analyzed with FlowJo version 10 software (TreeStar, Ashland, Oregon)
Cell population abundance	10 000 to 20 000 naïve ILC2s and 25 000 to 40 000 activated ILC2s were sorted per mouse. Purity was assessed by analyzing sorted cells for the same markers used for sorting. Purity greater than or equal to 90% was considered satisfactory.
Gating strategy	Relevant gating strategies shown in Figure 1, Figure 6, and Supplementary Figure 1 and 5. Briefly, all cells were first selected on an SSC-A/FSC-A scale based on known size and granularity of leukocytes and the lower limits was set to 50k. Subsequently, doublets that deviated from a linear increase were excluded on a FSC-H/FSC-A scale. For murine ILC2s, cells were gated as lineage-CD45+IL-7R+ and ST2+. for murine macrophages, cells were gated as CD45+CD11b+F4/80+. For human ILC2s, cells were gated as lineage-CD45+ CD127+ and CRTH2+. For both murine and human experiments, the boundaries between negative and positive populations were identified via isotype and FMO controls.

- [x] Tick this box to confirm that a figure exemplifying the gating strategy is provided in the Supplementary Information.
